# Human T Lymphotropic Virus Type 1 (HTLV-1): Molecular Biology and Oncogenesis

**DOI:** 10.3390/v2092037

**Published:** 2010-09-24

**Authors:** Priya Kannian, Patrick L. Green

**Affiliations:** 1 Center for Retrovirus Research, The Ohio State University, Columbus, OH 43210, USA; E-Mail: priya.kannian@cvm.osu.edu; 2 Department of Veterinary Biosciences, The Ohio State University, Columbus, OH 43210, USA; 3 Department of Molecular Virology, Immunology, and Medical Genetics, The Ohio State University, Columbus, OH 43210, USA; 4 Comprehensive Cancer Center and Solove Research Institute, The Ohio State University, Columbus, OH 43210, USA

**Keywords:** HTLV-1, leukemogenesis, cellular transformation, Tax, HBZ

## Abstract

Human T lymphotropic viruses (HTLVs) are complex deltaretroviruses that do not contain a proto-oncogene in their genome, yet are capable of transforming primary T lymphocytes both *in vitro* and *in vivo*. There are four known strains of HTLV including HTLV type 1 (HTLV-1), HTLV-2, HTLV-3 and HTLV-4. HTLV-1 is primarily associated with adult T cell leukemia (ATL) and HTLV-1-associated myelopathy/tropical spastic paraparesis (HAM/TSP). HTLV-2 is rarely pathogenic and is sporadically associated with neurological disorders. There have been no diseases associated with HTLV-3 or HTLV-4 to date. Due to the difference in the disease manifestation between HTLV-1 and HTLV-2, a clear understanding of their individual pathobiologies and the role of various viral proteins in transformation should provide insights into better prognosis and prevention strategies. In this review, we aim to summarize the data accumulated so far in the transformation and pathogenesis of HTLV-1, focusing on the viral Tax and HBZ and citing appropriate comparisons to HTLV-2.

## 1. General Background and Overview

Human T lymphotropic virus type 1 (HTLV-1) is a complex leukemogenic retrovirus with a single stranded positive sense RNA genome that expresses unique proteins with oncogenic potential. There are four known strains of HTLV, of which HTLV-1 and HTLV-2 are the most prevalent worldwide. HTLV-1 was originally identified in 1980 in a T cell line derived from a patient with cutaneous T cell lymphoma [1] and was also detected in adult T cell leukemia (ATL) patients [2,3]. Subsequently, HTLV-2 was identified in a cell line derived from a patient with a variant form of hairy T cell leukemia [4–6]. Since then, HTLV-2 has not been associated with leukemia/lymphoma; nevertheless, it has been associated with a few sporadic cases of neurological disorders [7]. HTLV-1 can infect T cells, B cells, monocytes, dendritic cells and endothelial cells with equal efficiency; yet, it can transform only primary T cells [8–11].

HTLV-1 is an enveloped virus that is approximately 100 nm in diameter. The inner membrane of the virion envelope is lined by the viral matrix protein (MA). This structure encloses the viral capsid (CA), which carries two identical strands of the genomic RNA as well as functional protease (Pro), integrase (IN), and reverse transcriptase (RT) enzymes. A newly synthesized viral particle attaches to the target cell receptor through the viral envelope (Env) and enters via fusion, which is followed by the uncoating of the capsid and the release of its contents into the cell cytoplasm. The viral RNA is reverse transcribed into double stranded DNA by the RT. This double stranded DNA is then transported to the nucleus and becomes integrated into the host chromosome forming the provirus. The provirus contains the promoter and enhancer elements for transcription initiation in the long terminal repeats (LTR); the polyadenylation signal for plus strand transcription is located in the 3′LTR [1].

**Figure 1.**
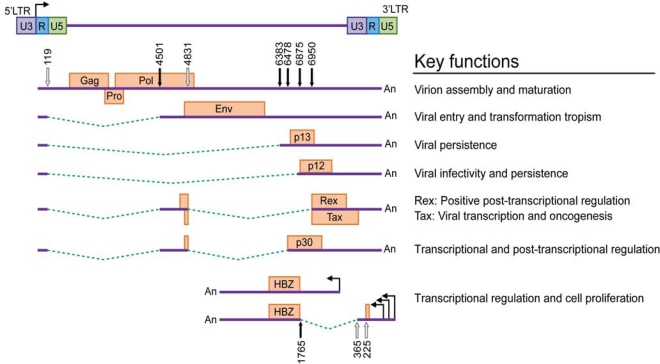
Structure of the HTLV-1 proviral genome and gene product key functions. The proviral DNA with the LTRs, and the unspliced, singly spliced and doubly spliced mRNA transcripts are shown to scale. The names of the gene transcripts are depicted inside each specific box (protein coding sequence). Solid lines indicate the exons and the dotted lines indicate the introns. Splice donor sites are indicated by open arrows and major splice acceptor sites are indicated by closed arrows. The numbers represent the nucleotide positions relative to the viral RNA. The general key functions for each of the genes at the protein level are listed to the right (see text for detail).

HTLV-1 is dependent on cellular factors for the initial rounds of transcription. The complex retroviral genome codes for the structural proteins Gag (capsid, nucleocapsid, matrix), Pro, polymerase (Pol) and Env from unspliced/singly spliced mRNAs [12–14] and regulatory and accessory proteins from alternatively spliced mRNA transcripts (Figure 1). The two regulatory genes *tax* and *rex* are encoded by open reading frames (ORF) IV and III, respectively, and share a common doubly spliced transcript. *Tax* is the transactivator gene, which increases the rate of viral LTR-mediated transcription [15–17] and modulates the transcription of numerous cellular genes involved in cell proliferation and differentiation, cell cycle control and DNA repair [18–23]. Tax has displayed oncogenic potential in several experimental systems [24–28] and is essential for HTLV-1 and HTLV-2-mediated transformation of primary human T cells [29–31]. Rex acts post-transcriptionally by preferentially binding, stabilizing and selectively exporting intron-containing viral mRNAs from the nucleus to the cytoplasm [32]. The accessory genes, *p12/p8* encoded by ORF I and *p30/p13* encoded by ORF II are dispensable in standard immortalization assays in culture but are essential for initiation of viral infection and the establishment of persistence in animal models [33–36]. p8 is a proteolytic cleavage product of the p12 parent molecule, whereas the p13 polypeptide, comprised of the carboxy terminus of p30, is expressed from a distinct mRNA. These accessory proteins may also play a role in gene regulation and contribute to the productive infection of quiescent T lymphocytes *in vitro* [37–40]. The minus strand of the proviral genome encodes several isoforms (generated from unspliced and spliced mRNAs) of the HTLV-1 basic leucine zipper factor (HBZ) [41]. HBZ interacts with cellular factors JunB, c-Jun, JunD, cAMP response element binding (CREB) and CREB binding protein (CBP)/p300 to modulate both viral and cellular gene transcription [42–44]. HBZ also plays a crucial role in T cell proliferation [45–47]. Among all the viral proteins, experimental evidence implicates Tax as the viral oncoprotein, but emerging data suggests a supporting role for HBZ in the oncogenic process.

## 2. Disease Association

HTLV-1 predominantly causes ATL and HTLV-1-associated myelopathy/tropical spastic paraparesis (HAM/TSP). There are five different clinical stages of ATL: asymptomatic carrier state, preleukemic state, chronic/smoldering ATL, lymphoma type and acute ATL [48–51]. The majority of the HTLV-1 infected patients are asymptomatic carriers who do not show any clinical symptoms. Even in the absence of symptoms, these individuals are capable of transmitting the virus to others. Approximately 1–2% of asymptomatic carriers progress to ATL over a 20–40 year period. HTLV-1 is less commonly associated with other disease conditions such as B cell chronic lymphocytic leukemia [52], chronic inflammatory arthropathy [53–55], HTLV-1 associated uveitis [56,57], T cell non-Hodgkin’s lymphoma [58,59], T-prolymphocytic leukemia, Sezary’s syndrome, small cell carcinoma, large granular lymphocytic leukemia (T-gamma lymphoproliferative disease) [60,61], dermatitis, lymphadenitis and Sjogren’s syndrome [62].

Although HTLV-2 initially was identified in a CD8^+^ T cell line derived from a patient with a variant form of hairy cell leukemia [4–6], there have been no subsequent reports of HTLV-2-associated neoplasms. However, there have been sporadic reports of HTLV-2-associated chronic encephalomyelopathy. The clinical symptoms presented are similar to those of HAM/TSP [63]. The prevalence of HTLV-2-associated myelopathy was reported to be 1% compared to 3.7% for HAM/TSP [64]. Although other neurological disorders have been reported, their clear association with HTLV-2 is hampered by confounding factors such as intravenous drug use or concomitant HIV infection [63]. To date, HTLV-3 and HTLV-4 have not been associated with any known clinical conditions.

## 3. Epidemiology

Approximately 15–25 million people worldwide are infected with HTLV-1 [62,65]. The virus is endemic in southwestern Japan [66], Africa [67,68], the Caribbean Islands [69] and South America [70] and is frequently found in Melanesia, Papua New Guinea [71], Solomon Islands and Australian aborigines [62]. HTLV-1 also is prevalent in certain populations in the Middle East [72] and India [73,74]. Of HTLV-1 infected patients, only 6.6% of males and 2.1% of females develop ATL [62]. HTLV-2 is more prevalent among intravenous drug users (IDUs), and is endemic among IDUs in the USA [75], Europe [76], South America [77] and southeast Asia [78]. HTLV-3 and HTLV-4 have been identified only in African primate hunters [79,80].

## 4. Viral transmission

Of the many possible routes of virus transmission, mother-to-child through breast feeding is the most predominant mode [81]. Transmission rates are 16% for children born to infected mothers, 27% for children nursed by infected mothers for more than three months and 5% for children nursed by infected mothers for less than three months [82,83]. Interestingly, about 13% of bottle-fed children also contract HTLV from their infected mother suggesting a route other than breast-feeding. The infants seroconvert within 1–3 years of age [83,84]. Sexual transmission rates are 60% for male to female, but only 0.4% for female to male transmission [85–87]. Predisposing factors associated with sexual transmission include the presence of genital ulcers, high viral loads and high antibody titers in the donor [86,87]. Among non-drug using sexual partners of IDUs, sexual transmission is a more common mode then parenteral transmission [88]. Among IDUs, blood and blood products are the most significant source of infection [89]. Approximately 12% of HTLV infections occur by blood transfusion. Unlike HIV-1, whole cell transfusion is required for transmission of the virus [90,91], with a seroconversion rate of approximately 50% [90,92]; however, the risk of transmission decreases markedly if the blood units are stored for more than six days before transfusion [91,93]. The development of HAM/TSP has been noted as early as six months after transfusion of an individual with infected blood [94]. In 1988, concerns about transmission of HTLV through blood components led to mandatory blood donor screening for HTLV resulting in a significant decrease in transmission via this mode.

Cell-free infection with HTLV-1 is very inefficient [95]; efficient transmission depends on cell-to-cell transfer through direct cell contact, polarization of the microtubule-organizing center (MTOC), which is triggered by Tax, and the formation of a virological synapse, which allows the entry of viral particles, viral proteins and genomic RNA into fresh target cells [96]. As with HIV-1 infection, dendritic cells (DCs) have been demonstrated to play a biphasic role in cell-to-cell transmission of HTLV-1. DCs can capture and transfer the virions to fresh T cells in a *trans* fashion or transmit *de novo* synthesized virions upon infection to fresh T cells in a *cis* fashion [97].

## 5. Viral persistence

Only about 1% of asymptomatic carriers progress to ATL, which occurs after about 2–4 decades of clinical latency. On the contrary, for HAM/TSP, disease progression can typically occur within a few years of infection [94,98]. In either case, the virus has co-evolved with its host to maintain lifelong persistence with an occasional exacerbation of pathological manifestations. HTLV-1 regulatory and accessory proteins, Rex, p12 and p30/p13 have been implicated to play a role in viral persistence. During the initial stage of infection, translation of Tax is favored over Rex due to a stronger Kozak sequence. Thus, the insufficient translation of Rex results in the export of only the doubly or completely spliced viral mRNAs, due to default splicing by the host cell machinery [99]. Eventually, accumulation of sufficient levels of Rex results in the expression of incompletely spliced mRNA in the cytoplasm, leading to the production of structural and enzymatic gene products and assembly of virus particles. Therefore, Rex is considered a positive regulator that controls the switch between early/latent and late/productive infection, which may help the virus avoid immune surveillance [32,99–101]. The expression of an accessory protein, p30, results in activation of the G2-M cell cycle checkpoint in Jurkat T cells, which suggests that p30 is involved in events that would promote early viral spread and T cell survival [102]. p30 also binds and retains doubly spliced *tax/rex* mRNA transcripts in the nucleus, thereby repressing viral gene expression and facilitating immune evasion [38,103]. Although p30 is dispensable for HTLV-1-mediated cellular transformation in cell culture, inoculation of rabbits with a p30-deficient virus revealed that p30 expression is required early in infection to sustain high viral loads and promote persistence in rabbits [33]. A recent report could not confirm the p30 ablation phenotype in HTLV-1 infected rabbits, but revealed its importance in viral persistence in macaques [36]. Thus, Rex is a positive post-transcriptional regulator, while p30 is a negative post-transcriptional regulator. Both viral proteins are maintained in a feedback loop to promote viral persistence and evasion of the host immune pressure [104].

A second accessory protein, p12, accumulates in the endoplasmic reticulum (ER) and Golgi compartments [105–107]. p12 interacts with interleukin-2 receptor β (IL-2Rβ) and IL-2Rγ chains leading to activation of the Janus kinase/signal transducer and activator of transcription 5 (Jak/Stat5) signal transduction pathway, and is required for efficient infection of quiescent primary T cells and syncytium formation [37,108–110]. The mechanisms by which p12 promotes immune evasion include interference with the presentation of major histocompatibility complex I (MHC class I) molecules by inducing the proteasomal degradation of the newly synthesized MHC molecules [107] and recruitment of p8 (the proteolytic cleavage product of p12 that facilitates the trafficking of the protein from the ER to the cell surface through the Golgi apparatus) to the virological synapse to down-regulate proximal signaling [40,111–113]. Another *in vitro* study demonstrated that p12 promoted cell-to-cell spread by inducing lymphocyte function-associated antigen 1 (LFA-1) clustering on T cells via calcium-dependent signaling [114]. *In vivo* animal models have established an essential role for p12 in persistent viral infection [34,36]. Valeri *et al.* have suggested that the lack of viral persistence of p12-deficient HTLV-1 in macaques is due to the inability of these viruses to efficiently infect DCs [36]. In the context of an infectious molecular clone, a third accessory protein, p13, was demonstrated to be dispensable for HTLV-1 infection and immortalization of primary T cells in culture [115]; whereas rabbits inoculated with a p13-deficient virus failed to induce a significant immune response and establish a persistent infection [116]. p13 mainly localizes in the mitochondria and suppresses tumor growth by interfering with *Ras* and *Myc* oncogenes [117,118]. Although p13 expression is not an apoptotic signal by itself, it sensitizes the cell to FasL and C-2 ceramide-induced apoptosis [118,119]. p13 alters the mitochondrial morphology by disrupting the inner membrane potential and calcium ion flux, and binds farnesyl pyrophosphate synthetase, an enzyme involved in post-translational prenylation of Ras [120]. Since Tax and p13 have opposing effects on apoptosis, the virus balances their functions to achieve two different scenarios. The first is to maintain a balance between the expression levels of Tax and p13 in order to regulate cell survival and proliferation of the infected cells leading to viral persistence. The second is to promote a cooperative effect, where p13 initially increases reactive oxygen species (ROS) in the mitochondria, which increases genetic instability or apoptosis in cells. Then, Tax promotes the selective growth and survival of these genetically unstable cells leading to the accumulation of DNA damage and progression towards neoplastic development [121].

## 6. Viral Transformation

Although HTLV-1 and HTLV-2 display differences in pathogenicity, both viruses can transform primary human T cells in cell culture. The precise mechanism by which these viruses transform T cells is not fully understood; however, a number of viral proteins have been implicated to play a role in HTLV- induced T cell transformation and pathogenesis. HTLV-1 and HTLV-2 exhibit differences in transformation tropism, where HTLV-1 preferentially transforms CD4^+^ T cells both *in vitro* and *in vivo* while HTLV-2 transforms CD8^+^ T cells in *in vitro* co-culture assays [122–125]. Tax-mediated transcription of HTLV-1 is significantly increased in CD4^+^ T cells as compared to CD8^+^ T cells, but the viral burden is higher in the latter [126,127]. The *in vivo* tropism of HTLV-2 appears to be less clear. Although Ijichi *et al.* have shown that HTLV-2 has a preferential tropism for CD8^+^ T cells *in vivo* [128], unlike HTLV-1, both CD4^+^ T cells and CD8^+^ T cells are equally susceptible to HTLV-2 infection and subsequent viral gene expression, with a greater proviral burden observed in CD8^+^ T cells [123,124,129]. In a quest to find the genetic determinant responsible for this differential transformation tropism, the first HTLV-1/2 recombinant viruses were generated and tested. Unexpectedly, results revealed that neither *Tax* nor the viral LTR sequences played a role [125,130]. Indeed, it was the viral envelope that conferred this distinct transformation tropism [130]. The viral envelope has two glycoproteins, surface component (SU) and transmembrane component (TM). SU binds to the cellular receptor, while TM triggers the fusion of the viral and cellular membranes, facilitating viral entry. Binding studies have supported the role of viral envelope in the distinct transformation tropism by showing that HTLV-1 binds to heparin sulfate proteoglycans (HSPG) on CD4^+^ T cells, while HTLV-2 binds to glucose transporter 1 (GLUT-1) on CD8^+^ T cells [131].

The viral transactivator protein, Tax, and the minus strand-encoded HBZ have been shown to play an essential role in HTLV-1 induced oncogenesis. Tax-induced activation of the nuclear factor κB (NF-κB) pathway [30] and the constitutive activation of the Jak/Stat pathway [132], and HBZ-induced activation or regulation of cellular factors like E2F1, JunB, c-Jun, JunD, CREB and CBP/p300 [112] have been implicated in transformation. The roles of Tax and HBZ in the induction of transformation are discussed in detail below. In addition, the accessory protein, p12, is also a modulator of T cell proliferation and immune function. p12 interacts with the 16 kDa subunit of the vacuolar ATPase, a complex important for the function of lysosomes and endosomes, and is implicated in transformation pathways [133,134]. Furthermore, p12 augments Ca^2+^ release from the ER via its ability to bind with calnexin and calreticulin, which regulate storage and release of Ca^2+^ from the ER, as well as with calcineurin, a calcium/calmodulin-dependent phosphatase, resulting in the activation of Nuclear Factor of Activated T Cells (NFAT). NFAT has a role in integrating calcium signaling with other signaling mechanisms in T cells [135–138]. Although p12 has been associated with proliferation, studies utilizing an infectious molecular clone indicated that abrogation of p12 message or protein had no effect on viral replication and immortalization of primary T cells *in vitro* [115].

## 7. Viral pathogenesis

The pathogenesis of ATL involves four stages: infection, polyclonal proliferation, clinical latency and tumorigenesis. HTLV-1 infects activated and dividing T cells with greater efficiency than quiescent T cells [139]. Env facilitates binding and entry into target cells. T cells become stimulated, which may be mediated by CD2/LFA-3, LFA-1/intracellular adhesion molecule (ICAM) and IL-2/IL-2R [140]. The activated T cells then form a pool of proliferating lymphoblasts. At this stage, the polyclonal population of cells is not leukemic. Indefinite T cell growth occurs in HTLV-1 infected individuals, but disease onset is seen only in a small percentage of these individuals. Tax and HBZ play a crucial role in the cell alteration process by triggering changes in a variety of intracellular signal transduction pathways, both by up-regulating and down-regulating viral and cellular gene expression in order to initiate neoplastic transformation [141,142]. The subsequent proliferation of the transformed T cells becomes IL-2 independent, which correlates with constitutive activation of the Jak/Stat pathways as well as decreased expression of src homology 2 (SH2)-containing tyrosine phosphatase-1 (SHP-1) protein, which regulates signaling from several hematopoietic surface receptors [143,144]. This transition usually correlates with significantly more rapid disease progression [145]. Upon infection of T cells, a period of clinical latency is observed in HTLV-1 carriers, which usually lasts 20 to 40 years. During this period, the viral genes are expressed at subdetectable levels to evade immune surveillance. HTLV-1 undergoes epigenetic silencing and also promotes chromosomal aberrations, leading to selection and evolution of monoclonal tumor populations. The degree of cytogenetic aberration is directly proportional to disease severity. The transactivation of proto-oncogenes such as *c-fos*, *egr*-1 and *egr*-2 by Tax may also contribute to leukemogenesis [146]. The development of tumors delineates the end of clinical latency and development of ATL in these patients.

Several animal models have been used to study HTLV-1 infection, persistence, and to some extent disease progression, although so far, these animal models have not been able to successfully mimic human ATL. To date, rabbits [147,148], rats [149,150], mice and non-human primates have been used as experimental models. Rabbits have been used more extensively because of ease of handling and development of a consistent infection that mimics the asymptomatic infection in humans. Although rabbits provide a good infection model, they are less helpful as tumorigenic models because they do not develop disease [151–155]. Certain strains of rats have been used to study HAM/TSP, although the neurodegenerative lesions are not very similar to those of humans. Results using rat models also suffer from variability depending on the rat strain employed [149,150]. Furthermore, in order to study tumorigenesis, the rats need to be immunodeficient [156]. Some non-human primates like Cynomolgus macaques and squirrel monkeys have been tested as animal models. Although these animals seroconvert variably, there are no typical signs of clinical disease [36,157,158]. Even though some investigators may argue that this subclinical state could mimic the human asymptomatic phase, the debate continues as to whether these models will mimic ATL if animals are maintained for longer periods; the long waiting time for such experiments make them unappealing. Lastly are the mouse models, where both xenograft and transgenic models have been utilized. Genetically normal and immunocompetent mice are not efficiently infected with HTLV-1 and they do not develop a natural course of illness. However, histologic analysis of xenograft models has shown ATL cells in various organs depending on the inoculation route. In addition, biochemical characteristics typical to HTLV-1 infection of humans including parathyroid hormone related protein (PTHrP) expression and increased serum IL-2Rα and β2-microglobulin levels correspond to increasing tumor burden in these mice [159–167]. Transgenic mice provide a good model to test the role of individual HTLV-1 genes in the pathogenesis of ATL. They also help in understanding the underlying relationship between the viral genes and their ability to cause unregulated cell growth [26,168–174]. The biggest caveat in such mouse models is the physiological relevance of these findings in the context of a completely active immune system. Humanized mouse models that are being used more extensively in HIV research thus far have been less attractive for HTLV-1 research because of the prolonged clinical latency period in the latter.

## 8. Role of Tax in HTLV-1 induced oncogenesis

Tax, a transactivator protein, triggers a plethora of events like cell signaling, cell cycle regulation and interference with checkpoint control and inhibition of DNA repair. Tax is expressed from a doubly spliced mRNA transcript. Although Tax shares the same mRNA transcript with Rex, translation of Tax is favored over Rex due to a stronger Kozak sequence. Tax made in the cytoplasm is translocated into the nucleus, where it binds to its response element and activates viral LTR-mediated transcription.

### 8.1. Effect of Tax on transcription factors

The Tax response element (TxRE) in the unique 3′ (U3) region of the 5′LTR is a 21-bp triple repeat, which contains an octamer motif TGACG(T/A)(C/G)(T/A) that is flanked by a G stretch at the 5′end and a C stretch at the 3′end. This octamer motif is homologous to the cAMP response element (CRE) 5′-TGACGTCA-3′ [175,176]. Tax does not have the specificity to directly bind TxRE DNA in the 5′LTR. *In vitro* biochemical studies have shown that Tax interacts with CRE binding/activating transcription factors (CREB/ATF) and forms a ternary complex with TxRE [177–183]. All of these proteins share common basic residues that facilitate DNA binding and include a basic leucine zipper domain (bZIP), which allows homo- and heterodimerization, which in turn is responsible for the ternary complex formation [184]. Tax interacts with the bZIP domain of CREB/ATF, enhances its dimerization, increases its affinity to the homologous octamer motif in the viral LTR and finally stabilizes the ternary complex by directly binding to the GC-rich flanking sequences [185–190]. Tax then recruits co-activators like CBP/p300 and p300/CBP-associated factor (P-CAF) for the initiation of transcription [191,192]. Tax binds to CREB/ATF and regulates LTR-mediated transactivation both positively and negatively. Tax also binds to co-activators of CREB – transducers of regulated CREB activity (TORCs), which facilitate the binding of Tax to CREB/ATF in a p300 and CBP-dependent manner [193,194]. Through its interactions with CREB/ATF, Tax represses a number of cellular genes including *p53*, *cyclin A* and *c-myb* [19,195,196]. Tax also activates promoters under the control of the serum responsive factor (SRF) responsive element (SRE) motifs via interactions with transcription factors associated with the SRF pathway. The main transcription factor under the transcriptional control of SRF is activator protein 1 (AP-1), which is triggered in response to various stimuli including cytokines, growth factors, stress signals, and oncogenes. AP-1 is either a homo- or heterodimeric complex of Fos (c-Fos, FosB, Fra1 and Fra2) and Jun (c-Jun, JunB and JunD) [146,197]. Moreover, Tax binds directly to SRF and to various members of the ternary complex factor (TCF) such as SRF accessory protein 1 (Sap1), Elk1, spleen focus forming virus (SFFV) proviral integration oncogene 1 (Spi1) and Ets1 [198–203]. These interactions increase the binding of SRF to multiple different SRE sequences located in the Fos/Jun promoters, thus occupying the CArG box (CC[A/T]_6_GG); then the TCF establishes a protein interaction with an upstream Ets box (GGA[A/T]). Once these complexes stabilize, Tax recruits the co-activators CBP/p300 and P-CAF to activate transcription [203]. Thus, Tax transactivation from CREB and SRF responsive sites involves its interaction with transcription factors by enhancing DNA binding, altered site selection and co-activator recruitment.

### 8.2. Tax and T cell transformation

Apart from activating viral gene transcription, Tax induces various cellular functions in the HTLV-1 infected cells and thus, renders them susceptible to viral persistence and thereby initiates neoplastic transformation. NF-κB is a key player in Tax-induced T cell transformation. The NF-κB family of transcription factors includes five structurally related members – RelA, RelB, c-Rel, NF-κB1 (p50/p105) and NF-κB2 (p52/p100) [204–206]. The precursor proteins, p105 and p100, are proteolytically cleaved to the mature p50 and p52 forms. These proteins form various dimeric complexes in the cytoplasm. In naïve T cells, the dimers are trapped by inhibitory IκB proteins such as p105, p100, IκBα, IκBβ and IκBγ. IκBs mask the nuclear localization signal (NLS) of the NF-κB factors by physical interaction [204,205]. Upon activation of the T cells by an appropriate stimulus, IκB kinase (IKK) phosphorylates IκB inhibitors, thereby triggering their ubiquitination and subsequent proteasomal degradation. This event leads to the exposure of the NLS and the eventual translocation of the NF-κB factors to the nucleus, where they transactivate or repress target genes bearing a κB enhancer [204,205,207]. The NF-κB family plays a crucial role in immune functions such as innate and adaptive responses, survival of lymphocytes and lymphoid tissue development [206]. Therefore, any aberrant NF-κB activation could lead to the genesis of cancer, especially hematologic malignancies such as leukemia, lymphoma and myeloma [208]. NF-κB functions via two signaling pathways – classical and alternate. Both pathways regulate overlapping but distinct cellular genes. The classical pathway is activated by inflammatory cytokines, genotoxic stress, antigens and toll-like receptor (TLR) stimulation, which results in degradation of IκB inhibitor and the translocation of p50/RelA complex into the nucleus. The alternate pathway is activated by a subset of tumor necrosis factor (TNF) family members such as CD40L, lymphotoxin-β, B cell activating factor belonging to the TNF family (BAFF), receptor activator for NF-κB ligand (RANKL) and TNF-like weak inducer of apoptosis (TWEAK). Activation of the alternate pathway results in the processing of p100/RelB into p52/RelB and the translocation of the latter to the nucleus [209].

In naïve T cells, the activity of NF-κB pathways is tightly controlled. NF-κB is transiently activated upon immune stimulation, but constitutively activated in HTLV-1 infected T cells [210–213]. Constitutive activation of NF-κB is mediated by Tax and is essential for the induction of T cell transformation. Mechanistically, Tax binds to IKKγ (NF-κB essential modulator, NEMO) and activates the IKK complex [214,215]. Tax also binds to transforming growth factor – β activated kinase 1 (TAK1), a mitogen activator protein-3 kinase (MAP3K), and stimulates IKK activity [216]. Tax undergoes post-translational modifications including phosphorylation, acetylation, sumoylation and ubiquitination [217–223]. Of these, ubiquitination is crucial for binding to NEMO and is dependent on E2 ubiquitin-conjugating enzyme, Ubc13 [221]. NEMO-related protein (NRP/Optineurin) binds to Tax and positively modulates the ubiquitination of Tax resulting in activation of the NF-κB pathway [224]. Tax expressed by HTLV-1 activates both the classical and alternate pathways of NF-κB by binding simultaneously to the IKK complex and NF-κB2/p100 leading to IKKα-mediated p100 phosphorylation and its subsequent cleavage into p52 [225]. HTLV-2 Tax can activate the classical pathway at levels comparable to that of HTLV-1 Tax, but cannot activate the alternate pathway because the former cannot interact with p100 [226]. Dependence on NF-κB activation by Tax for the immortalization of T cells both *in vitro* and *in vivo* has been demonstrated by several groups [29,30,227]. Tax-mediated NF-κB activation stimulates a number of cytokines and their corresponding receptors such as IL-2/IL-2R, IL-9, IL-13, IL-15/IL-15R, IL-21/IL-21R, IL-8, CCL2, CCL5, CCL22, CCR9, CXCR7, CD40, OX40/OX40L and 4-1BB/4-1BBL [228–245]. Approaches to block NFκB using drugs or peptide inhibitors have resulted in tumor cell regression in animal models [227].

### 8.3. Tax and pathogenesis

Tax has two main functions in the pathogenesis of HTLV-1. First is constitutive cell cycle progression and the second is resistance to apoptosis. Tax induces cell cycle progression by protein-protein interaction and transcriptional regulation of cell cycle-associated proteins. Specifically, Tax stimulates the transition of cells from G1 to S phase through the hyper-phosphorylation of Rb and activation of E2F transcription factors and by the induction of cyclin D2 and cyclin-dependent kinase 6 (CDK6) via the NF-κB pathway (both classical and alternate). In addition, Tax activates CDK4/CDK6 through the direct interaction with CDK4, CDK6 and CDK inhibitors like CDKN1A, p16^INK4A^ and p15^INK4B^ [246–255]. Thus, Tax induces the expression of cell cycle regulators in an NF-κB-dependent manner and subsequently activates them in an NF-κB-independent manner. Tax also influences transformation and regulates apoptosis in T cells by activation of the phosphatidyl inositol 3 kinase (PI3K)/Akt pathway. Mechanistically, Tax frees a catalytic p110α subunit of the PI3K complex from an inhibitory p85α subunit by binding directly to the latter [256]; Tax induces the RelA-mediated sequestration of p300 from the promoters of phosphatase and tensin homolog (PTEN) and SH2 domain-containing inositol phosphatase - 1 (SHIP-1), thereby down-regulating their expression [257]; Tax influences a number of factors that affect the PI3K/Akt pathway including mammalian target of rapamycin (mTOR), AP-1, NF-κB, CREB/ATF, β-catenin, and hypoxia inducible factor – 1 (HIF-1) [256,258–261]. Tax induces anti-apoptotic proteins such as Bcl-xL, survivin, cFLIP, xIAP, cIAP1 and cIAP2 in an NF-κB-dependent manner [262–267]. Both the classical and alternative NF-κB pathways play a positive role in the inhibition of apoptosis in HTLV-1-infected T cells.

Tax exhibits its oncogenic potential by both clastogenic DNA damage and aneuploidic effects [112,184,268,269]. Tax promotes clastogenic DNA damage by repressing the expression of DNA polymerase β, which is involved in base excision repair, nucleotide excision repair, and repression of human telomerase reverse transcriptase (hTERT), thus subverting Ku80 activity (a protein essential for DNA repair) [269]. All of these mechanisms result in reduced protection from double stranded DNA breaks as well as telomere extension, which could be the reason for end-to-end fusion of chromosomes observed in HTLV-1-infected cells. During DNA damage, the complex DNA damage response (DDR) signaling molecules such as ATM, ATR and DNA-PK kinases become activated and orchestrate DNA repair [268,270,271]. Tax can induce checkpoint activation and cell cycle arrest at the G1 phase by inducing p27/kip1 and p21/waf1 [272]. This function of Tax appears to be paradoxical to its transforming activity. Nevertheless, the constitutive activation of the DDR pathway and the checkpoint adaptation facilitating the proliferation of T cells with DNA damage causes genetic instability and ultimately evolution of ATL clones. Tax causes aneuploidy by interacting with proteins that monitor chromosomal segregation during mitosis through the following mechanisms: induction of supernumerary centrosomes and multipolar mitosis via interactions with Tax-1 binding protein-2 (TAX1BP2) and Ran/TC4-binding protein (RanBP1); unscheduled degradation of securin and cyclin B1 by interacting with CDC20-associated anaphase-promoting complex (APC); and impairment of mitotic spindle assembly checkpoint protein 1 (MAD1) activity, an integral function of the mitotic spindle assembly checkpoint (SAC) [112]. Tax also plays a key role in promoting oncogenesis by abrogating the function of the tumor suppressor gene, *p53*. The biological activity of p53 is central to the integrity of a cell in that any loss of its function either due to mutation or inactivation increases the chance of genetic instability leading to oncogenesis. The subcellular localization and phosphorylation status of p53 are critical for its function. In a majority of HTLV-1 infected cell lines, p53 function is impaired although the protein itself is sufficiently expressed and stable. Two mechanisms have been identified for the abrogation of p53 function by Tax. First, it was demonstrated that the expression of Tax-1 or Tax-2 impaired the functionally relevant phosphorylation of p53 at serines 15 and 392; Tax activation of the NF-κB pathway was essential for this activity [273,274]. Second, it was demonstrated that Tax competes with p53 for the binding with CBP/p300, which results in the decreased ability of p53 to activate cell cycle control genes [275,276].

A PDZ binding motif (PBM) or domain, which is comprised of a four amino acid sequence at the C-terminus of HTLV-1 Tax, has been implicated in T cell proliferation and transformation. The PDZ domain is named after the first identified PDZ-containing proteins, post-synaptic density protein (PSD-95), *Drosophila* discs large protein (DLG) and epithelial tight junction protein (Zonula Occludens-1). The PDZ domain is commonly used in eukaryotic cells to recruit and organize proteins to sites of cellular signaling. Using both virus gene knockout and knockin approaches, Xie *et al.* investigated the role of the Tax PBM in both cell culture and infected animals. These authors demonstrated that the PBM of HTLV-1 Tax significantly increased HTLV-1-induced primary T cell proliferation *in vitro*. Moreover, HTLV-1 proviruses containing a deletion in this four amino acid motif had severely attenuated infectivity and persistence *in vivo* [277]. HTLV-2 Tax does not contain this PBM domain. Interestingly, a chimeric HTLV-2 virus containing the HTLV-1 Tax PBM significantly increased primary human T cell proliferation *in vitro,* thus lending further support that this domain plays a key role in Tax pathogenic activity[277]. The three PDZ-containing proteins in humans are hDLG, membrane associated guanylate kinase (MAGUK) with inverted orientation – 3 (MAGI-3) and precursor of interleukin–16 (pro-IL-16), all of which have been implicated in tumor suppression or cell cycle regulation, and have been demonstrated to interact with Tax PBM [278–281]. HTLV-1 Tax PBM competes with the adenomatous polyposis coli (APC) tumor suppressor protein for the hDLG binding domain [282]; PBM competes with another tumor suppressor, phosphatase with tensin homology mutated in multiple advanced cancers (PTEN/MMAC) for binding to MAGI-3 [283]; and PBM also interacts with the first PDZ domain of pro-IL-16 [280], all of which result in the reversal of cell cycle arrest in G0/G1 phase induced by hDLG. Using a panel of Tax-1 and Tax-2 mutants, the PBM has been demonstrated to enhance micronuclei induction in transfected cells, which probably plays a role in the genomic instability caused by Tax [277,284,285]. PBM also has been shown in other oncogenic viruses including human papillomavirus and adenovirus, which points toward a possible mechanism for PBM and PDZ-containing proteins in cellular transformation and pathogenesis by tumorigenic viruses [209,286,287].

## 9. Role of HBZ in HTLV-1-induced oncogenesis

HBZ is encoded by an mRNA transcribed from the minus strand of the proviral genome. Transcription from the antisense strand of HTLV-1 was first reported in 1989 [288]. Almost a decade later, the viral protein HBZ was detected in HTLV-1 transformed cell lines. HBZ was identified as a binding partner of CREB-2 by yeast two-hybrid assays [41]. *Hbz* transcription is driven by a TATA-less promoter in the 3′ end of the proviral genome activated primarily by Sp1 [289]. HBZ is expressed from three mRNA transcripts, unspliced form (*usHbz*) and two spliced forms (*sHbz*) – major and minor, as identified by 5′ rapid amplification of cDNA ends (RACE) [46,290,291]. The *sHbz* transcripts have multiple initiation sites in the unique 5′ (U5) and R regions of the 3′LTR, whereas the *usHbz* initiates from within the *tax* gene. The TxRE in the 3′LTR functions as the promoter enhancer element for *Hbz* transcription, although its function is much weaker compared to the 5′LTR TxRE [289,292]. This provides one explanation why the gene expression of *tax* and *Hbz* are inversely proportional during the course of HTLV-1 infection and disease progression. There are marked differences between the unspliced and spliced HBZ isoforms both at the RNA and protein levels (Table 1). *Hbz* transcripts are expressed at relatively constant levels in ATL cells, regardless of the *tax* expression levels [293]. Total *Hbz* transcripts detected in an infected individual are directly proportional to the proviral load, probably due to their dependence on Sp1 for transcription [46]. The difference between *sHbz* and *usHbz* transcripts is the presence of the first exon in the former. This region corresponds to a small portion of the Rex-responsive element (RxRE) in the sense strand. In the antisense strand, this region forms a variable stem loop structure that might interact with host factors to induce the proliferation of ATL cells in an IL-2-independent manner [46,289]. Saito *et al.* have reported a correlation between the *Hbz* transcript levels and the severity of HAM/TSP, which suggests a possible role for HBZ in the pathogenesis of HAM/TSP [293].

Western blot analyses have shown the expression of sHBZ protein in almost all ATL cell lines. The HBZ protein contains three domains – activation, central and basic leucine zipper (bZIP) [41,295]. HBZ binds to cellular factors like c-Jun, JunB, JunD, CREB-2 and CREB via the bZIP domain [41,42,44]. The bZIP and the activation domains are involved in the activation of JunD [45], increase of hTERT [296] and the binding with the p65 subunit of NF-κB [297], thereby inhibiting the classical pathway of NF-κB activation. HBZ protein has been shown to localize in the nucleoli with a speckled staining pattern by immunohistochemistry. The integrity of the amino acid sequence of HBZ must be maintained for this speckled pattern to be observed [295]. This nuclear localization signal is located in the central domain and is comprised of three regions: two regions rich in basic amino acids and one DNA binding domain. HBZ interacts with the CBP/p300 KIX domain and the 26S proteasome through the activation domain [298]. HBZ interacts with CREB-2 (ATF-4) through the bZIP domain, which abolishes the binding of CREB-2 to TxRE in the 5′LTR and, in turn, results in the down-regulation of Tax-mediated viral transcription in an HBZ dose-dependent manner [41,299]. HBZ inhibits the transcriptional activity of cellular factors by localizing in the heterochromatin and also by sequestering JunB into nuclear bodies [300,301]. HBZ disrupts the basal transcription of both HTLV-1 and cellular promoters by attenuation of AP-1 activation (Fos/Jun dimers) via degradation of c-Jun in a ubiquitination-independent manner and also by directly interacting with the 26S proteasome, thereby causing proteasomal degradation of c-Jun [42,45]. In addition, HBZ interacts with JunD to activate cellular genes including hTERT, which activates the telomerase in cell mitosis, a critical late event in tumor progression that indicates a role for HBZ in the development and maintenance of leukemic cells [296]. It is interesting to note that JunD is primarily a growth suppressor that functions by interacting with the tumor suppressor, menin [302]. However, in the presence of HBZ, JunD forms homodimers with HBZ and thereby increases its transcriptional and transforming activity. Recently, both isoforms of HBZ have been shown to hetero-dimerize with MafB via their bZIP domains and abrogate the DNA binding activity of MafB. *MafB* is a transcriptional factor that is responsible for lineage-specific hematopoiesis. Additional investigations are required to delineate the effect of this suppression [303].

### HBZ and pathogenesis

Although no antibody responses against HBZ have been detected so far from HTLV-1-infected carriers [304], recent evidence has shown that HBZ is immunogenic *in vitro*. When exposed to DCs, HBZ was processed and presented in the context of HLA-A*0201. HBZ peptides induced specific cytotoxic T lymphocytes (CTL), but they failed to lyse HLA-A*0201-positive HTLV-1-infected T cell lines and ATL cells. This could be because the amount of HBZ protein expressed by HTLV-1 infected cells is not sufficient to be recognized by the HBZ-specific CTLs. HBZ-specific CTLs are detected in very sparse numbers in ATL patients and healthy carriers [305].

Kinetic analyses of gene expression levels in HTLV-1 proviral plasmid over-expression studies in cell culture revealed that *Hbz* mRNA levels are significantly lower than *tax/rex* mRNA levels [306]. In the context of an infectious molecular clone, like other accessory gene products, *Hbz* was dispensable for immortalization of primary human T cells *in vitro*. Stable T cell clones generated with *Hbz* defective proviruses produced significantly higher levels of p19 Gag protein indicating increased Tax-mediated viral gene expression [307]. Stable HBZ expression promoted the IL-2-independent survival of Kit-225 cells (a leukemic T cell line that is solely dependent on exogenous IL-2 for survival) [46] and increased the proliferation of Jurkat T cells [47]. Knockdown of HBZ expression by shRNA decreased cell proliferation in ATL cell lines, HTLV-1 transformed cell lines, and newly immortalized primary T cells [47]. *Hbz* RNA, specifically a stem loop structure near the amino terminus of the transcript, and not the protein, increased T cell proliferation by increasing the transcription of the *E2F1* gene [46]. HBZ binds to the Rel homology domain of the p65 subunit of NF-κB and degrades p65, thus inhibiting the classical NF-κB pathway. This function is mechanistically tailored by the increase of E3 ubiquitin ligase, PDLIM2 (PDZ and LIM domain containing protein 2) [297,308]. NF-κB functions through a classical and an alternate pathway and both have distinct regulatory functions. The alternate pathway is critical for cellular transformation, while both the pathways regulate anti-apoptotic genes differentially in lymphoma cell lines. HBZ enhances the expression of PDLIM2, which suppresses Tax-mediated tumorigenicity by promoting the degradation of Tax. Thus, HBZ suppresses Tax both at the RNA and protein levels.

Recently, Polakowski *et al.* have demonstrated that siRNA-mediated knockdown of CBP/p300 or a truncated form of CBP/p300 containing the KIX domain abrogated the expression of Dickkopf-1 (Dkk-1), a Wnt signaling inhibitor in HBZ transfected cells [309]. Dkk-1 has been shown to play an important role in the development of bone lesions in multiple myeloma. Lytic bone lesions are also a symptom of ATL. *DKK-1* mRNA levels were positively correlated with the expression of *Hbz* in HTLV-1 infected cell lines. Interestingly, Tax represses the expression of Dkk-1 consistent with the opposing forces of Tax and Hbz within the cell. Taken together, in the late stages of ATL, when Tax is repressed, HBZ supports cell proliferation and the maintenance of the leukemic cell.

*In vivo* studies in rabbits revealed a significant reduction in proviral load and attenuated antibody response against the viral proteins correlating with the loss of HBZ function [299]. Proviral load was decreased by 5-to-50-fold as early as two weeks post infection in these animals. Kinetic analyses of viral gene expression confirmed the *in vitro* data that *Hbz* mRNA levels were low early after infection and then increased to a stable plateau. This trend was similar to that of the proviral loads and was the reverse of the *tax/rex* mRNA levels [307]. Thus, HBZ plays an important function in viral persistence. There was an increase in CD4^+^ T cells in the spleens of transgenic mice expressing HBZ under the control of the mouse CD4 promoter/enhancer [46]. Another study with NOD/SCID^γchain−/−^ mice showed that HBZ knockdown in a transformed T cell line significantly reduced tumor formation and organ infiltration [47]. Together, these two studies confirm that HBZ is essential for promoting T cell proliferation.

## 10. Combined mechanisms of Tax and HBZ in HTLV-1-induced oncogenesis

**Figure 2.**
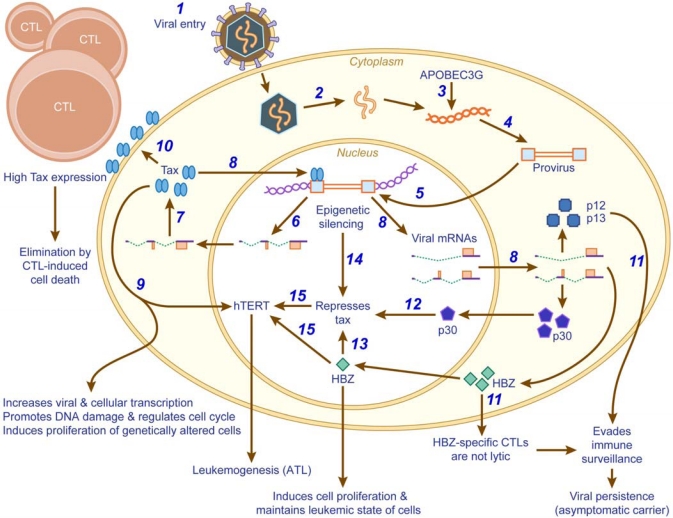
**A schematic model illustrating the possible mechanisms by which HTLV-1 succumbs to and evades the host immune system.** The sequence of events is labeled with Arabic numerals. 1: attachment and entry of a virion into a target cell; 2: the capsid uncoats releasing the viral RNA; 3: low frequency editing of the genomic RNA by APOBEC3G, one possible mechanism for epigenetic silencing; 4: reverse transcription into double-stranded DNA; 5: proviral integration into the host chromosome; 6: initial transcription and export of completely/doubly spliced viral mRNA by host cellular factors; 7: favorable translation of Tax due to a strong Kozak sequence; 8: Tax transactivation of the viral LTR to promote viral gene expression; 9: Tax increases cellular transcription, promotes DNA damage, regulates cell cycle and induces proliferation of genetically altered cells. Tax also increases hTERT function initially to induce transformation of virus infected cells, but subsequently down-modulates hTERT to accumulate chromosomal rearrangements and maintain transformation; 10: high expression of Tax by virus infected cells results in their elimination by Tax-specific CTL induced cell death; 11: expression of viral accessory proteins like p12 and p13 facilitates viral persistence in the host. Additionally, the sparsely elicited HBZ-specific CTLs do not lyse ATL cells due to the low HBZ protein expression levels in all ATL cell lines and HTLV-1 transformed cell lines. This helps in immune evasion and viral persistence; 12: the accessory protein, p30, translocates to the nucleus and forms ribonucleoprotein complexes with Rex to retain *tax/rex* mRNA in the nucleus, and thus represses the expression of Tax; 13: HBZ represses Tax at the transcriptional level by competing for CREB-2 and CBP/p300, and at the protein level by enhancing the expression of PDLIM2. Furthermore, HBZ complements for the reduced activity of Tax by activating cellular factors to induce transformation and proliferation of genetically unstable cells; 14:epigenetic silencing mechanisms also result in decreased Tax expression levels facilitating viral persistence; 15: reduced Tax and increased HBZ levels reactivate hTERT, which is a key event in the progression of ATL.

A number of mechanisms involving Tax and HBZ have been demonstrated to play a role in HTLV-1-induced oncogenesis (Figure 2). Tax is indispensable for the transformation process induced in HTLV-1 infected cells. However, once transformed, these cells do not require Tax to maintain their leukemic state [184,310]. Tax is detected only in about 40% of ATL patients [311]. In approximately 10% of ATL cases, genetic changes in *tax* have been documented; DNA methylation of *tax* has been shown in another 15% of ATL patients; and deletion of the 5′LTR and promoter region has been shown in an additional 27% of ATL cases [311–316]. In addition, p30 has been shown to repress viral replication at the post-transcriptional level by binding to and retaining *tax/rex* mRNA in the nucleus [38,103]. By suppressing Tax protein expression, p30 attenuates HTLV-1 transcription. More recently, it was reported that p30 and the positive post-transcriptional regulator Rex form ribonucleoprotein complexes specifically on *tax/rex* mRNA [317]. Together, these data suggest that p30 may govern the switch between viral latency and replication.

In HTLV-1, the 3′LTR is identical to the 5′LTR. Both carry the TxRE sequences that have the enhancer elements for transcription. However, the enhancing elements of the antisense transcript are much weaker than the sense transcript. This could be part of the reason why *Hbz* is detected during the later stages of HTLV infection, unlike *tax/rex* and *gag/pol*, which are expressed at high levels during the early stages of infection. Expression of HBZ is correlated positively with proviral loads and negatively with *tax/rex* and *gag/pol* expression [307]. HBZ expression also is correlated positively with disease severity in ATL and HAM/TSP, suggesting a role in pathogenesis [46,293]. HBZ was detected in all ATL cells, including those that lacked Tax expression. As mentioned above, the absence of Tax in these cells is attributed to genetic changes in the *tax* gene and/or the 5′LTR. Neither the *Hbz* gene nor the 3′LTR are affected by any of these epigenetic silencing mechanisms. Miyazaki *et al.* showed that in one ATL case, the polyadenylation site of *Hbz* gene was deleted. However, the *Hbz* gene utilized a downstream polyadenylation signal for transcription [316].

Tax initially increases hTERT expression facilitating lifespan extension and immortalization of cells. Once the virus establishes persistence, Tax represses hTERT via an E-box present in the hTERT promoter probably through competition for CBP/p300, and thus favors accumulation of chromosomal rearrangements and then the transformation of the infected cells towards a malignant phenotype. Subsequent epigenetic silencing of Tax in the leukemic cells reactivates hTERT, which in turn, stimulates the proliferative potential of the infected cells leading to ATL. The reactivation of hTERT is a key event in the induction of ATL progression, which is probably tailored by HBZ [302].

Another possible mechanism that could cause epigenetic silencing has been elucidated recently by Fan *et al.* [318]. They showed that HTLV-1 is prone to RNA editing by human APOBEC3G during reverse transcription, although the editing is at a low frequency. The authors have attributed the nonsense mutations in the plus strand of the proviral DNA coding for *env, tax, p13* and *p30* to be responsible for the reduced viral gene expression in these cells. The generation of nonsense mutations does not affect the *Hbz* gene, which codes from the minus strand. Moreover, there are very few mutation sites in the opposite strand of the HBZ coding region. Therefore, the functional activity of HBZ is not affected. It also was shown that these mutations occur both in ATL cells and HTLV-1 infected cells from asymptomatic carriers, indicating that these mutations do not occur during oncogenesis but are present even during the carrier state. The mutations were present in the leukemic cells and not in the non-leukemic HTLV-1 infected cells, suggesting that there is a selection for these cells during leukemogenesis, which likely favors the virus by promoting immune evasion [318]. Evidence from another group suggests that the 5′LTR deletion occurred before integration in 8/12 ATL cases and after integration in 4/12 ATL cases. This deletion was detected in 3.9% of the carriers and in 27.8% of ATL patients. In these defective proviruses, the second exons of the *tax, rex* and *p30* genes were frequently deleted, and thus Tax-mediated activation of NF-κB and CREB pathways was abrogated. In all these cases, the coding sequences of the *Hbz* gene were intact [316].

Taken together, the possible mechanism of HTLV-1-induced oncogenesis suggests that Tax is expressed initially to induce transformation and cell proliferation. Tax-specific CTLs mediate the death of virus infected cells. Thus, the virus is forced to down-regulate Tax expression to below detectable levels by the expression of HBZ in order to persist in the host. Since HBZ-specific CTLs do not lyse ATL cells and HBZ can promote and maintain the leukemic state of these cells, this mechanism ought to be favored by the virus in order to evade immune surveillance and continue to persist in the host. However, why it would be in the best interest of the virus to cause cancer, which is a dead end situation for itself, although in a very small percentage of infected individuals, is still an unanswered question.

## 11. Conclusions

HTLVs are complex retroviruses with unique proteins that have oncogenic potential. There are four known strains of HTLV, HTLV-1, HTLV-2, HTLV-3, HTLV-4; only HTLV-1 and HTLV-2 have been consistently associated with disease in humans. HTLV-1 mainly causes ATL and HAM/TSP. HTLV-2 is not etiologically oncogenic and has been associated with some neurological disorders. HTLV-3 and HTLV-4 were identified in African primate hunters without any subsequent etiological disease associations so far.

A complete understanding of the functions of the viral genes would give insights into the pathogenic mechanisms by which HTLV-1 induces oncogenesis. In this review we summarize the data published so far in this field with pertinent comparisons to HTLV-2, the non-leukemic counterpart. Like simple retroviruses, HTLV-1 expresses structural and enzymatic proteins for its assembly and maturation, and for entry into new target cells. HTLV-1 also expresses regulatory and accessory proteins that are essential for viral persistence, immune evasion and ultimately, leukemogenesis. Although, the exact mechanisms and pathways have not been fully elucidated, much is known thus far. For instance, Tax is expressed in the early stages of infection to establish viral transcription and induce T cell transformation by regulating cellular transcription factors, inducing G1 to S phase transition and DNA damage resulting in genetic instability, and promoting proliferation of genetically altered (leukemic) cells. Subsequently, HBZ suppresses Tax expression to evade immune elimination by Tax-specific CTLs, and also complements for Tax to support proliferation; it provides a second oncogenic signal required for the maintenance of the leukemic cell. Further investigations are warranted to determine the additional events in the minor population that progresses to ATL.

## Figures and Tables

**Table 1.** Structural and functional differences between the unspliced and spliced Hbz.

**Structure/Function**	**usHBZ**	**sHBZ**
**RNA**		
Transcription initiation site [47,293,294]	Within the *tax* gene	Multiple sites in the U5 and R region of the 3′LTR
Weak promoter enhancement by TxRE [47,293,294]	?	Yes
mRNA transcription efficiency [47,293,294]	0.25X	1X
Promotes T cell proliferation [46,289]	Weak	Strong
**Protein**		
Half-life of the protein [47,293,294]	Short	Long
Detection levels in ATL cell lines and HTLV-1 infected cells	Low	High
Inhibition of AP-1 transcription [42,45]	Weak	Strong
Delivery of c-Jun to proteasomal degradation [42,45]	Strong	Weak
